# Supplementary material for: Supercooling of the A phase of 3He
Source: Nat Commun. 2023 Jan 10;14:148. doi: 10.1038/s41467-022-35532-7 (PMC9832038; doi:10.1038/s41467-022-35532-7)
Supplement: Supplementary file 1 — Supplementary Information [file 41467_2022_35532_MOESM1_ESM.pdf]

Supplementary Information: Supercooling of the A phase of ^{3}He

Y. Tian[1], D. Lotnyk[1], A. Eyal[1,2], K. Zhang[3,4], N. Zhelev[1], T.S. Abhilash[1,5], A. Chavez[1], E.N. Smith[1], M. Hindmarsh[3,4], J. Saunders[6], E. Mueller[1], and J.M. Parpia[1]

[1]*Department of Physics, Cornell University, Ithaca, NY, 14853, USA*

[2]*Physics Department, Technion, Haifa, Israel*

[3]*Department of Physics and Astronomy, University of Sussex, Falmer, Brighton BN1 9QH, UK*

[4]*Department of Physics and Helsinki Institute of Physics, PL 64, FI-00014 University of Helsinki, Finland*

[5]*VTT Technical Research Centre of Finland Ltd, Espoo, Finland*

[6]*Department of Physics, Royal Holloway University of London, Egham, TW20 0EX, Surrey, UK*

November 28, 2022

Supplementary Note 1: Experimental details

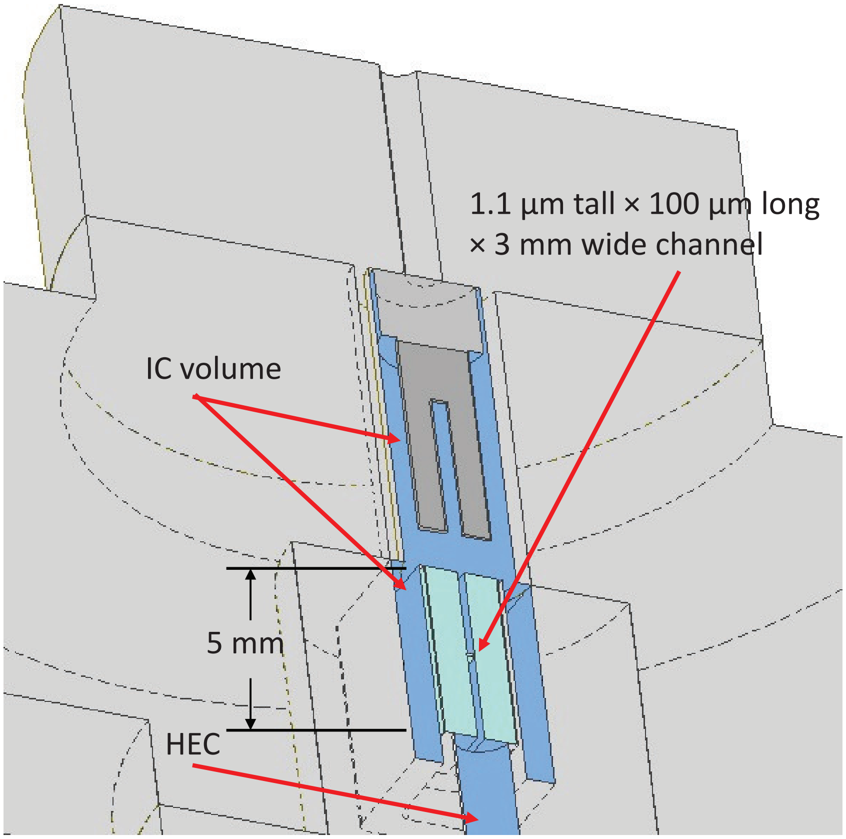

Supplementary Figure 1: Schematic of the IC. The assembly comprising the silicon and glass channel is shown as light blue and is 5 mm in length. The channel separating the IC (top) and HEC (region below the channel) is in the form of a wide septum with a letterbox shape 100 μm long $\times$ 1.1 μm tall $\times$ 3 mm wide, and is shown in cross-section with the channel width not visible here. Most of the open volume resides in the bulk liquid surrounding the channel holder and around the quartz fork located above the channel. There are regions where close fitting coin-silver components that comprise the cell structure are separated by gaps of order 25 μm that may promote the A phase. All surfaces of the coin-silver components are as-machined metal.

The measurement of the AB transition occurs independently (at low pressures in pressure varied transitions and below $\sim$ 24 bar under constant pressure cooling) in the small volume isolated chamber (IC). The IC is connected to a chamber containing a sintered silver heat exchanger (HEC) through a 1.1 μm high, 3 mm wide, and 100 μm long channel with 200 μm tall $\times$ 3 mm wide $\times$ 2.45 mm long "lead-in" sections at either end. The 1.1 μm height section appears to be filled with the B phase at all temperatures and pressures to the left of the red contour highlighted in Fig. 5 of the main paper. To the right of this line the channel isolates the two chambers, and the transitions occur independently. To the left of this line, the B phase nucleated in one chamber will grow into the other. The physical arrangement is illustrated in Fig. 2 of the main paper. The channel (Supplementary Figure 1, shown in light blue) was nanofabricated in 1 mm thick silicon, capped with 1 mm thick sodium doped glass, anodically bonded to the silicon[1] and then glued into a coin silver carrier.

The volume of the IC is estimated to be 0.14 $\pm$ 0.02 cm^3, and the area of all wetted surfaces in the IC was estimated to be 14.5 $\pm$ 0.5 cm^2. In comparison, the HEC had a volume of 0.72 $\pm$ 0.1 cm^3 and a surface area of 3.5 $\pm$ 0.5 m^2 due to the heat exchanger.

Supplementary Note 2: Landau Ginzburg theory

Superfluid ^{3}He has a complex order parameter which can be represented by a 3×3 matrix $A_{\alpha j}$, where α corresponds to a spin angular momentum index, and j an orbital angular momentum index: both take on 3 possible values: x, y, z. The 2×2 gap matrix is $\Delta = \sum_{\alpha j} i\sigma_\alpha \sigma_y A_{\alpha j} p_j$, where the momentum $\mathbf{p}$ can be taken to lie on the Fermi surface. The A-phase corresponds to a rotation of $A_{\alpha j} = f_\alpha(\delta_{jx} + i\delta_{jy})$, for which the orbital angular momentum points in the $\hat{z}$ direction, and the gap matrix has a node in that direction. If one ignores dipolar interactions, f is a completely arbitrary vector of fixed magnitude. The B phase corresponds to a rotation of $A_{\alpha j} \propto \delta_{\alpha j}$. This is an isotropic fully gapped state with the spin and orbital angular momenta entangled with one-another.

In equilibrium the order parameter minimizes a free energy $\Omega = \int (f_{\text{bulk}} + f_{\text{grad}}) d^3 r$. We neglect dipolar terms, which are on a smaller energy scale. Near T_c, the resulting bulk free energy density is written[2–5]

$$\begin{aligned} f_{\text{bulk}} &= \alpha \text{Tr}(AA^\dagger) + \beta_1 |\text{Tr}(AA^T)|^2 + \beta_2 |\text{Tr}(AA^\dagger)|^2 \\ &\quad + \beta_3 \text{Tr}(AA^T)(AA^T)^* + \beta_4 \text{Tr}(AA^\dagger)^2 + \beta_5 \text{Tr}(AA^\dagger)(AA^\dagger)^*. \end{aligned}$$

The coefficients are

$$\alpha = \frac{1}{3} N(0) \left(\frac{T}{T_c} - 1 \right) \tag{1}$$

$$\beta_j = \beta_0 \tilde{\beta}_j \tag{2}$$

$$\beta_0 = -\frac{N(0)}{(\pi k_B T_c)^2} \frac{7}{240} \zeta(3) \tag{3}$$

We use transition temperatures $T_c(p)$ tabulated by Greywall[6,7], converted to the PLTS scale using

the procedure in Ref. 8. The density of states at the Fermi Surface is $N(0)$, and T_c is the normal-superfluid transition temperature.

At T_c, the strong coupling β-coefficients are $\tilde{\beta}_j = \tilde{\beta}_j^{\mathrm{WC}}(p)+\Delta\beta_j(p)$, and we use the $\Delta\beta_j(p)$'s which are tabulated by Regan, Wiman, and Sauls (RWS)[9], who calculated the values from first principles. The weak coupling β's are $\tilde{\beta}_1^{\mathrm{WC}} = 1, \tilde{\beta}_2^{\mathrm{WC}} = \tilde{\beta}_3^{\mathrm{WC}} = \tilde{\beta}_4^{\mathrm{WC}} = -2, \tilde{\beta}_5^{\mathrm{WC}} = 2$. We rescale the RWS pressures by a factor of 1.01 so that the polycritical point occurs at the experimental $p_{PCP} = 21.22$ bar.

We estimate the temperature dependence of the strong-coupling coefficients by taking $\tilde{\beta}_j^{SC} = \tilde{\beta}_j^{\mathrm{WC}}(p) + (T/T_c)^{\eta(p)}\Delta\beta_j(p)$. We choose $\eta(p)$ in order to correctly match the experimental A-B transition temperature[7], which are well approximated by $T_{AB}/T_c(p) \approx 1 - 0.174(p - p_{PCP})/\mathrm{bar}$. As explained below, the A-B equilibrium line is at $\beta_1 + \beta_3/3 - 2\beta_5/3 = 0$. We therefore take

$$\eta(p) = -\frac{\log -(\Delta\beta_1 + \Delta\beta_3/3 - 2\Delta\beta_5/3)/(\beta_1^{\mathrm{WC}} + \beta_3^{\mathrm{WC}}/3 - 2\beta_5^{\mathrm{WC}}/3)}{\log(1 - 0.174(p - p_{PCP})/\mathrm{bar})}. \tag{4}$$

This results in $\eta = 1.79, 1.58, 1.26$ at $p = 19, 24$, and 29 bar. Previous works[6,9] which used $\eta(p) = 1$, find a somewhat shifted A-B line. Some of the quantities we calculate are quite sensitive to the location of the transition, necessitating the extra empirical parameter.

We have also explored other approaches to correct for this shift in the A-B line. Supplementary Note 6 presents one of those, where we simply rescale the parameters, $\tilde{\beta}_j^{SC} = \tilde{\beta}_j^{\mathrm{WC}}(p) + \alpha(p)\Delta\beta_j(p)T/T_c$. We have also repeated the analysis with alternative strong-coupling parameters in the literature [5,6]. All models give similar predictions, once the location of the equilibrium AB

transition line is corrected.

The gradient terms in the free energy are typically written as

$$f_{\rm grad} = \sum_{\mu j k} K_1 |\nabla_k A_{\mu j}|^2 + K_2 \nabla_j A^*_{\mu j} \nabla_k A_{\mu k} + K_3 \nabla_j A^*_{\mu k} \nabla_k A_{\mu j} \tag{5}$$

with $K_1 = K_2 = K_3 = K$, and

$$K = \frac{7\zeta(3)}{60} N(0)\xi_0(p). \tag{6}$$

It is convenient to scale the energy and order parameter in order to make the equations dimensionless. Thus we take $A = A_0 \tilde{A}$, $f = |\alpha| A_0^2 \tilde{f}$, and choose A_0 such that $\beta_0 A_0^4 = \alpha A_0^2$. We also rescale lengths by the temperature dependent correlation length $\xi = \xi_{GL}/\sqrt{1 - T/T_c}$, to arrive at a dimensionless equation. Here the Ginzburg-Landau coherence length is $\xi_{GL} = \sqrt{7\zeta(3)/20}\xi_0$.

For the bulk A and B phases we take $\tilde{A}_{\alpha j} = a\delta_{\alpha j}(\delta_{jx} + I\delta_{jy})/\sqrt{2}$ and $\tilde{A}_{\alpha j} = a\delta_{\alpha j}/\sqrt{3}$. These ansatzes yield free energy densities of the form $\tilde{f} = -a^2 + a^4\beta_\Phi$, where $\Phi = A, B$ denotes the phase, and the quartic coefficients for each phase are $\beta_A = \beta_{245}$ and $\beta_B = \beta_{12} + \beta_{345}/3$. Here we use the convention that appending indices yields the sum, so $\beta_{234} \equiv \beta_2 + \beta_3 + \beta_4$. Minimizing with respect to a, gives $\tilde{f} = -1/(4\beta)$. The equilibrium AB transition occurs when these energies are equal to one-another, ie $\beta_1 + \beta_3/3 - 2\beta_5/3 = 0$.

We use a finite difference approach to find the structure of the AB domain wall, taking $\tilde{A}$ to be a function of one spatial coordinate, x. We minimize the free energy with the constraint that $\tilde{A}$ takes on its bulk A and bulk B form at $x = 0$ and $x = L$. For the minimization we use a Broyden, Fletcher, Goldfarb, Shanno quasi-Newton method, discretizing space into 200 segments, each of

which is 5% of the correlation length. Supplementary Figure 2 illustrates how the components of $\tilde{A}$ vary in space, as well as the local energy density. We define the position of the domain wall by the location of the peak in the local energy density, x_p. The surface tension is then extracted as $\sigma = f - f_A x_p - f_B(L - x_p)$. Here f_A, f_B are the bulk free energies.

We similarly find the surface energy associated with bulk A or B phase contacting the chamber's wall: τ_A, τ_B. We consider two extreme boundary conditions: minimal and maximal pairbreaking. The former models specular scattering of quasiparticles from the wall[10], and the latter corresponds to quasiparticle retroreflection[11].

For maximal pairbreaking, the order parameter simply vanishes at the wall. For minimal pairbreaking the component of the order parameter normal to the surface vanishes, $\sum_j A_{\alpha j} n_j = 0$, where n_j are the components of the normal vector to the wall. The other components have zero slope, $\sum_j n_j \nabla_j A^{\perp}_{\alpha i}$. Here $A^{\perp}_{\alpha i} = A_{\alpha i} - n_i \sum_j (A_{\alpha j} n_j)$ is the component of A which is perpendicular to the surface.

For minimal pairbreaking one can align the A phase order parameter so that $\tau_A = 0$. While there is some temperature and pressure variation, $\tau_B \approx 0.8\sigma$. For maximal pairbreaking, $\tau_A \approx 2\sigma$ and $\tau_B \approx 2.5\sigma$. The fact that $\tau_A < \tau_B$ for maximal pairbreaking can be traced to the contribution of the various gradient terms in Eq. (5).

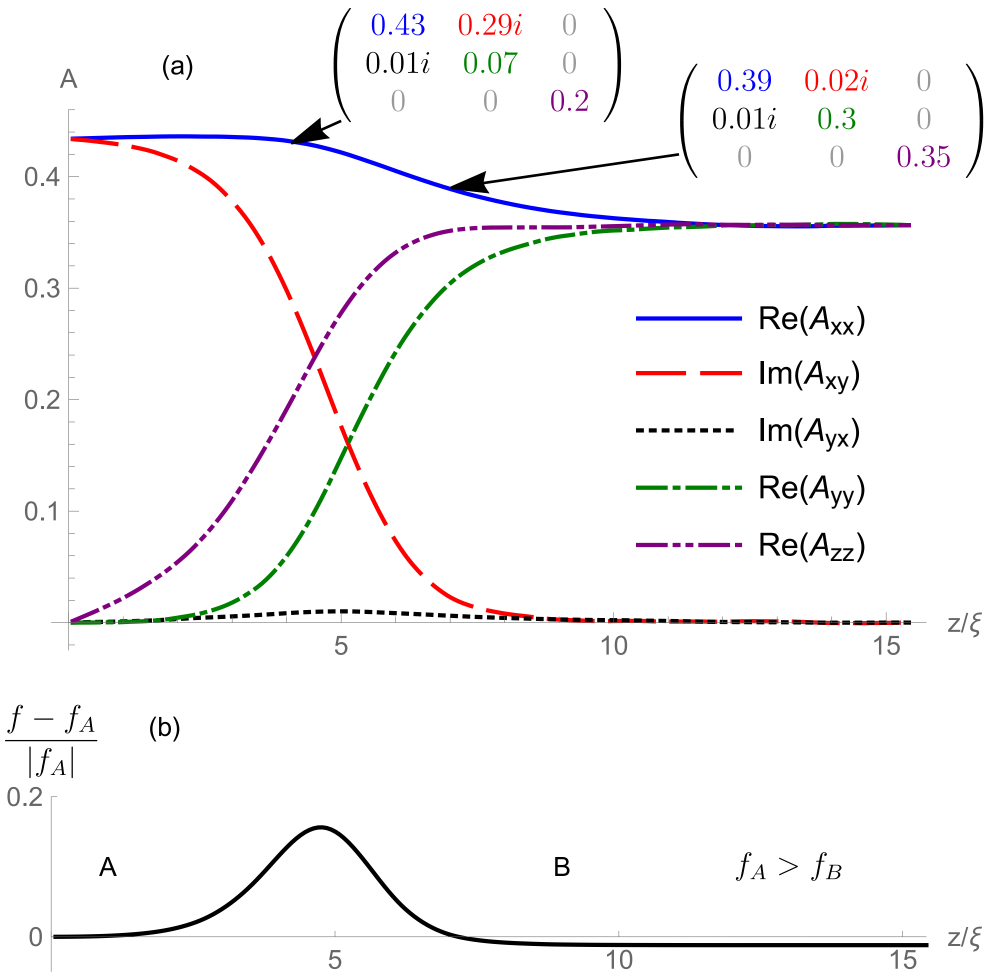

Supplementary Figure 2: **Spatial dependence of the order parameter in a A-B domain Wall.** (a) Components of the order parameter $A_{\alpha j}$ in a domain wall between the A phase (left) and B phase (right). The order parameter is constrained to its bulk A and B values at the edges. Magnitudes are scaled to dimensionless units, as described after Eq. (6). (b) Local free energy density, f, in this domain wall, as compared to the energy density of the A-phase. Here $T = 2.1$ mK and $p = 24$ bar, and the temperature dependent Ginzburg-Landau coherence length is $\xi = 39$ nm. For these parameters, the free energy of the B-phase is somewhat lower than the A-phase. The surface tension σ is calculated by integrating the the curve in (b), accounting for the offset between f_A and f_B.

Supplementary Note 3: Stability of a domain wall across an orifice

In our experiment we observe path dependent hysteresis in the A to B phase transition. The simplest model which could account for this observation involves positing that the cell contains a large number of small chambers connected to the main fluid via small orifices. Leggett and Yip[12] described such chambers as "lobster pots," and used them to explain related hysteretic phenomena. The orifices have a distribution of sizes, and we posit that each of the chambers contain B-fluid. The bulk fluid is A. The stability of an AB domain wall across such an orifice was theoretically studied by Viljas and Thuneberg[13]. As we review below, each AB boundary is only stable if the orifice size W is smaller than some critical value $W < W_c$, where W_c depends on pressure and temperature. If $W > W_c$, and we are in the part of the phase diagram where the A-phase is stable, the bulk A-phase will rush into the chamber, destroying the B-seed. Conversely, in the B-region of the diagram, the B-phase will rush out, converting the bulk to B. The same argument can be used to analyze the ability of the 1.1 μm letterbox channel to prevent the B-phase from propagating from the HEC to IC.

In this model, the system's memory comes from the distribution of chambers which contain B, and the catastrophe line is set by the largest orifice of a B-chamber. We emphasize that this chamber model is merely intended to be an easily analyzed toy. It is likely that the true B-seeds involve frustrated A textures, whose modeling is complex. These may be bulk textures, or they may involve boundaries. Similar energetic considerations will be at play – regardless of the microscopics. The relevant scales are the free-energy difference between the two phases, the domain

wall surface tension, and the size of the texture.

The chamber model seems particularly relevant for describing the sinter in the HEC. Even there, however, the geometry is likely more complicated. As argued by Leggett and Yip[12], the openings of the chambers can be tapered, or have some other asymmetry, which will lead to quantitative discrepancies.

We treat the A-B domain wall as an elastic membrane. Consequently, the equilibrium domain wall will be bowed, with the phase with higher free energy on the inside. Balancing forces implies that in equilibrium its mean curvature is $\kappa = \delta f/(2\sigma)$, where δf is the difference in free energy density between the fluids on each side of the wall, and σ is the surface tension[14]. The mean curvature is the arithmetic mean of the two principle curvatures: spherical and cylindrical surfaces of radius R respectively have $\kappa = 1/R$ and $\kappa = 1/(2R)$.

Supplementary Figure 3 shows a number of simple geometries for a domain wall spanning a constriction. Panels (a) and (b) show the case where the constriction can be modeled as a hole in a flat sheet. In (a) the A phase has lower free energy than B, and the domain wall bows into the B region. Equating forces at the edge of the aperture requires $\tau_B - \tau_A \leq \sigma\cos(\theta)$, where τ_B and τ_A are the surface energy associated with each phase, and θ is the angle that the surface makes with the walls – which also corresponds to half the angle subtended by the opening, as measured from the center of the arc which defines the domain wall. Consequently, the domain wall is stable if $W \leq 2R\sin(\theta)$. Panel (b) shows the case where B has a lower free energy than A. Since $\tau_A < \tau_B$, surface energies are never relevant and the domain wall is stable if $W < 2R$.

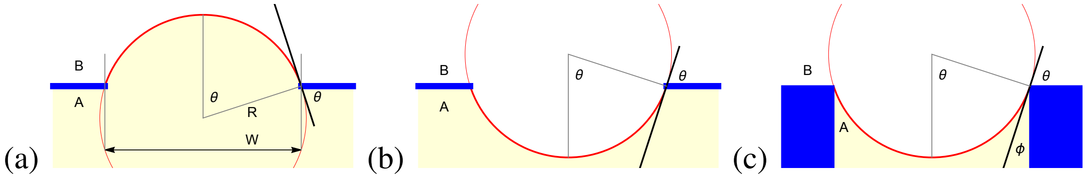

Supplementary Figure 3: **Geometry of AB phase boundary.** Within the elastic model of the AB boundary, an equilibrium domain wall forms a surface of constant mean curvature. Three geometries are depicted: (a) and (b) show the case where the domain wall is pinned by a circular hole or a long slit. In (a) the free energy density of the A phase is less than that of the B phase, while (b) shows the opposite. Panel (c) depicts the case where the A phase is contained in a narrow channel.

Panel (c) of Supplementary Figure 3 shows the case where the constriction has parallel sides. This models the letterbox channel connecting the HEC and IC. Because $\tau_A < \tau_B$, such a configuration is never stable with B inside the chamber and A outside. The illustrated configuration is stable as long as $\tau_B - \tau_A \geq \sigma \cos(\phi)$. Here the angle that the domain wall makes with the surface, ϕ, is complementary to the angle subtended by the domain wall, θ. Thus the domain wall is stable if $W \leq 2R(\tau_B - \tau_A)/\sigma$. Specializing to the case of a slit, where $R = 1/(2\kappa) = \sigma/\delta_f$, this expression simplifies to $W \leq 2\delta\tau/\delta f$, which does not depend on the AB surface tension. Equality coincides with the equilibrium A to B phase transition in a thin channel: $W\delta f = 2\delta\tau$.

The pressure and temperature dependence of the contact angle is illustrated in Supplementary Figure 4 for both minimal and maximal pairbreaking boundary conditions. The A-phase surface energy vanishes for minimal pairbreaking, resulting in a significantly smaller contact angle. Due to the behavior of the strong-coupling parameters, the contact angle is slightly larger at high temperatures and high pressures.

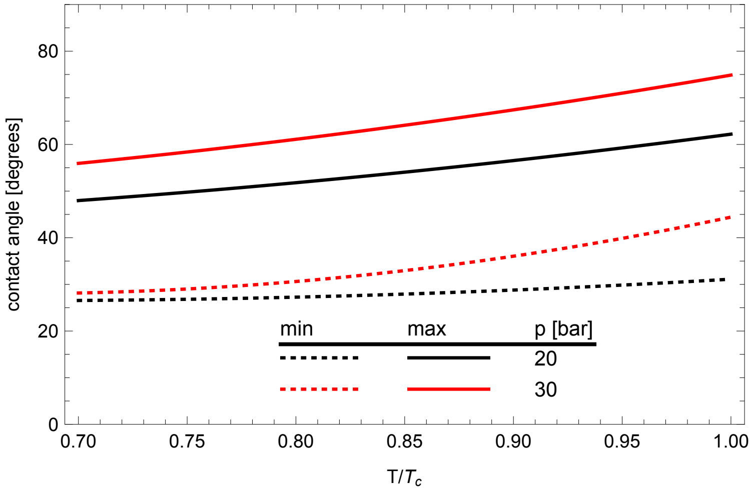

Supplementary Figure 4: **Contact angle of an AB domain wall with a surface.** Solid lines: Maximal pairbreaking. Dotted lines: Minimal pairbreaking. Black: $p = 20$ bar, Red: $p = 30$ bar.

Previous experiments have directly tested aspects of this elastic model of the AB phase boundary[15–17], including measuring equilibrium contact angles, surface tensions, and surface energies. A number of theoretical works have also addressed the issue[18–20].

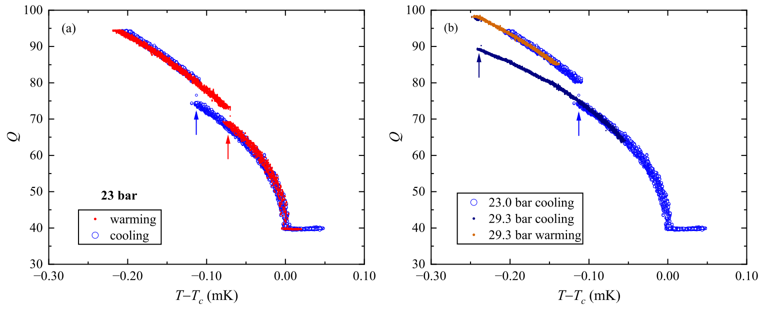

Supplementary Figure 5: Q **vs T in HEC.** (a) Q vs T of the HEC fork (blue open circles-constant pressure cooled at 23 bar) (red dots-warming). (b) Black points - depressurized at constant Q from 29.3 bar to 23 bar, then cooled at constant pressure - extend the supercooling beyond that accessible at constant pressure (compare Blue and Black arrows). Only the part of the trajectory where $p = 23$ bar is shown. Orange points show part of the data obtained while warming in the B phase after depressurization.

Supplementary Note 4: Comparing constant pressure and pressure varied A-B transitions

In the main document we established that the degree of metastability of the A-phase depended on the trajectory taken through the phase diagram. Here we compare two trajectories. Supplementary Figure 5(a) shows the quality factor of the HEC fork during cooling (blue) and heating (red) at 23 bar. Supplementary Figure 5(b) shows the same cooling data, and overlays the Q measured while first cooled at 29.3 bar to 2.15 mK, and then depressurized to 23 bar (in the A phase) followed by cooling at constant pressure. Data is only shown for the part of the trajectory at 23 bar. In all cases the A-B transition is visible as a discontinuity in Q. The degree of metastablity is enhanced by transiting through the 29.3 bar A-phase; however, the Q in both phases is consistent, showing that the additional supercooling cannot be due to a pressure lag, heating effects, or a significant change in the properties of the bulk fluid.

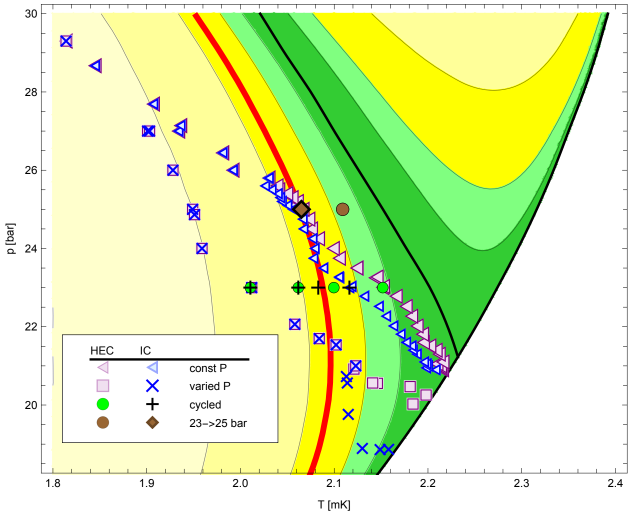

Supplementary Figure 6: **Curvature of stable domain walls, using maximum pairbreaking.** Contours show $\kappa_A/\sin(\theta_A)$ and κ_B in the A and B portions of the phase diagram, using maximal pairbreaking boundary conditions to calculate $\sin(\theta_A)$. All values coincide with those of Fig 5 of the main text. Here, because of the change in boundary conditions, the contours in the A-phase are shifted to slightly higher pressures.

Supplementary Note 5: Comparison of boundary conditions

To highlight the role of boundary conditions, we reproduce the analysis used for Figs. 5 and 6 of the main paper, but using maximal pairbreaking boundary conditions. As seen in Supplementary Figure 6, the maximal pairbreaking contours of $\kappa/\sin\theta$ are shifted to slightly higher pressures compared to the minimal pairbreaking shown in Fig. 5 of the main paper. This results in a larger value of Γ, plotted in Supplementary Figure 7. Given that our model predicts $\Gamma = 1$, we conclude that minimal pairbreaking represents a better fit for the surfaces of the silver sinter.

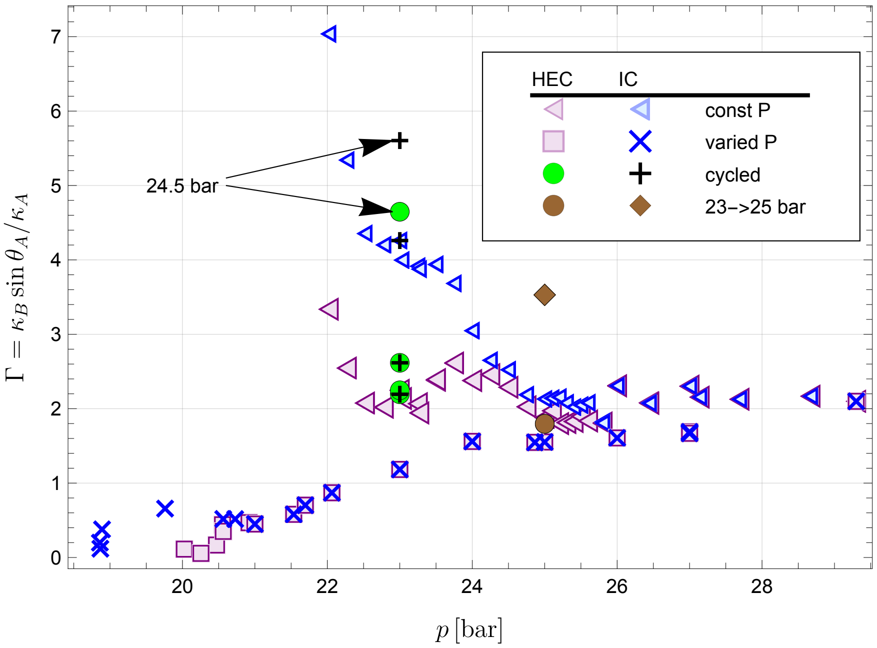

Supplementary Figure 7: **Pressure dependence of Γ at catastrophe point for maximal pairbreaking boundary conditions.** Ordinate, Γ is the ratio of the domain wall curvature at catastrophe point, κ_B, to the largest scaled curvature traversed in A-phase, $\kappa_A/\sin(\theta_A)$. The contact angle θ_A depends on the boundary condition: here we use maximal pairbreaking. The abscissa shows the pressure at the observed A-B transition. This graph should be compared to Fig. 6 of the main text, which used minimal pairbreaking (specular scattering) at boundaries.

Supplementary Note 6: Alternate renormalization of the Ginzburg-Landau parameters

In Supplementary Note 2, we explain how we introduce a phenomenological scaling function $\eta(p)$ which allows the Landau-Ginzburg theory to correctly model the pressure dependence of the AB phase transition line. Here we present an alternative approach, where we simply rescale the Regan, Wiman, and Sauls coefficients[9], taking $\tilde{\beta}_j^{SC} = \tilde{\beta}_j^{\rm WC}(p) + \alpha(p)\Delta\beta_j(p)T/T_c$.

We again use the approximation that $T_{AB}/T_c(p) \approx 1 - 0.174(p - p_{PCP})/\text{bar}$. Using the fact that the A-B transition occurs when $\beta_1 + \beta_3/3 - 2\beta_5/3 = 0$, We conclude

$$\alpha(p) = -\frac{\beta_1^{\rm WC} + \beta_3^{\rm WC}/3 - 2\beta_5^{\rm WC}/3}{\Delta\beta_1 + \Delta\beta_3/3 - 2\Delta\beta_5/3}\frac{1}{1 - 0.174(p - p_{PCP})/\text{bar}} \tag{7}$$

which yields $\alpha = 1.03, 0.98, 0.96$ at $p = 19, 24, 29$ bar.

Figure 8 shows the results of using this alternative approach to modeling the experiment. While the renormalization scheme causes small changes in the results, our general conclusions are unchanged. This concordance gives us confidence in our results.

The most notable difference between the renormalization schemes is in the cycled paths, which are cooled at 23 bar, transit to higher pressure, and then continue cooling at 23 bar (shown as green circles and black "+" marks). In Fig. 8 the outliers are somewhat less extreme than in the main text. The model dependence of Γ for these points indicates that one should be cautious about ascribing too much significance to them.

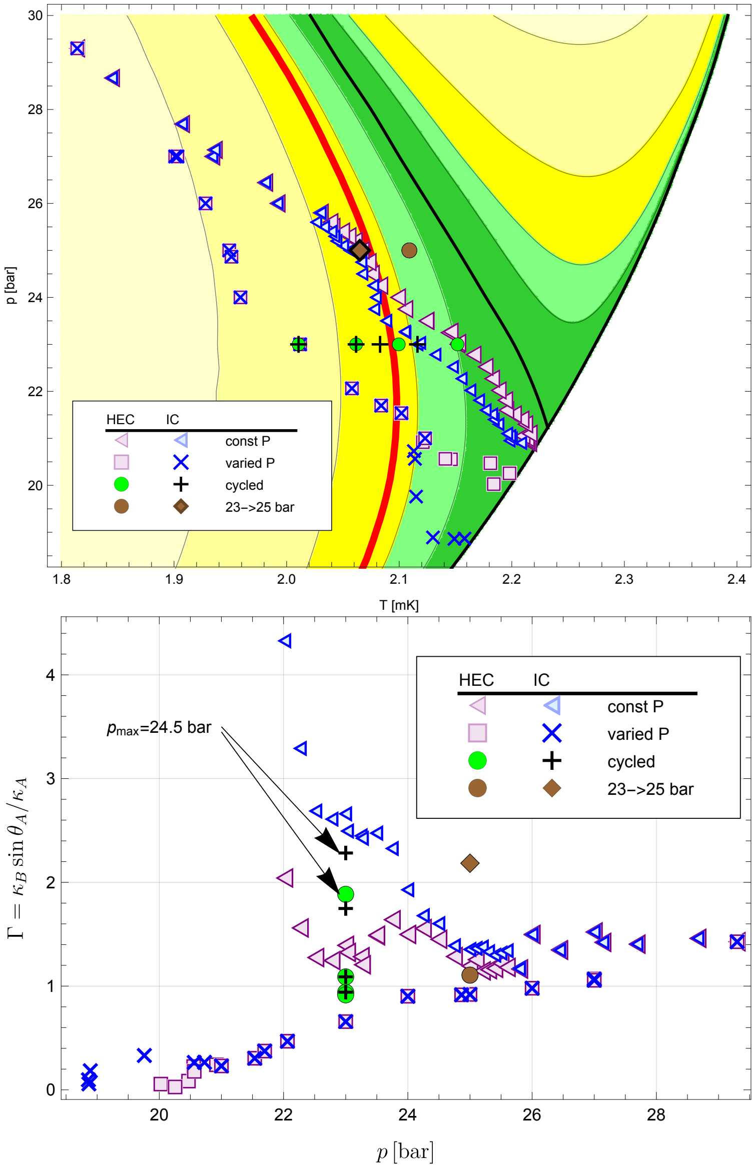

Supplementary Figure 8: **Results from alternate renormalization of strong coupling parameters.** Graphs correspond to Figs. 5 and 6 from the main text, but with the alternate model for temperature dependence of the Landau Ginzburg parameters from Supplementary Note 6:.

References:

1. Zhelev, N. *et al.* Fabrication of microfluidic cavities using Si-to-glass anodic bonding. *Rev. Sci. Instrum.* **89**, 073902 (2018). URL http://dx.doi.org/10.1063/1.5031837.

2. Vollhardt, D. & Woelfle, P. *The Superfluid Phases Of Helium 3* (Dover, Mineola New York, 2013). URL https://doi.org/10.1201/b12808.

3. Wiman, J. J. & Sauls, J. A. Superfluid phases of ^{3}He in nanoscale channels. *Phys. Rev. B* **92**, 144515 (2015). URL https://link.aps.org/doi/10.1103/PhysRevB.92.144515.

4. Wiman, J. J. & Sauls, J. A. Strong-coupling and the stripe phase of 3-He. *J. Low Temp. Phys.* **184**, 1054–1070 (2016). URL https://doi.org/10.1007/s10909-016-1632-7.

5. Choi, H., Davis, J. P., Pollanen, J., Haard, T. M. & Halperin, W. P. Strong coupling corrections to the ginzburg-landau theory of superfluid ^{3}He. *Phys. Rev. B* **75**, 174503 (2007). URL https://link.aps.org/doi/10.1103/PhysRevB.75.174503.

6. Wiman, J. J. & Sauls, J. A. Superfluid phases of ^{3}He in nanoscale channels. *Phys. Rev. B* **92**, 144515 (2015). URL https://link.aps.org/doi/10.1103/PhysRevB.92.144515.

7. Greywall, D. ^{3}He specific heat and thermometry at millikelvin temperatures. *Phys. Rev. B* **33**, 7520–7538 (1986). URL http://dx.doi.org/10.1103/PhysRevB.33.7520.

8. Tian, Y., Smith, E. N. & Parpia, J. Conversion between $^3\mathrm{He}$ melting curve scales below 100 mK. *Journal of Low Temperature Physics* **184**, 1573–7357 (2022). URL https://doi.org/10.1007/s10909-022-02721-z.

9. Regan, R. C., Wiman, J. J. & Sauls, J. A. Vortex phase diagram of rotating superfluid $^3\mathrm{He}-\mathrm{B}$. *Phys. Rev. B* **101**, 024517 (2020). URL https://link.aps.org/doi/10.1103/PhysRevB.101.024517.

10. Ambegaokar, V., deGennes, P. G. & Rainer, D. Landau-Ginsburg equations for an anisotropic superfluid. *Phys. Rev. A* **9**, 2676–2685 (1974). URL https://link.aps.org/doi/10.1103/PhysRevA.9.2676.

11. Sauls, J. A. Surface states, edge currents, and the angular momentum of chiral p-wave superfluids. *Phys. Rev. B* **84**, 214509 (2011). URL https://link.aps.org/doi/10.1103/PhysRevB.84.214509.

12. Leggett, A. J. & Yip, S. K. Nucleation and growth of $^3\mathrm{He} - \mathrm{B}$ in the supercooled A-phase. In Pitaevskii, L. P. & Halperin, W. P. (eds.) *Helium Three*, no. 26 in Modern Problems in Condensed Matter Sciences, chapt. 8, 523–707 (Elsevier, Amsterdam, 1990), third edn.

13. Viljas, J. & Thuneberg, E. Stability of A–B phase boundary in a constriction. *Physica B: Condensed Matter* **329-333**, 86–87 (2003). URL https://doi.org/10.1016/s0921-4526(02)01889-6.

14. Isenberg, C. *The science of soap films and soap bubbles* (Dover Publications, Mineola, NY, 1992).

15. Bartkowiak, M. *et al.* The Unique Superfluid ^{3}He A-B Interface: Surface Tension and Contact Angle. *Journal of Low Temperature Physics* **126**, 533–538 (2002). URL `https://doi.org/10.1023/a:1013758916085`.

16. Bartkowiak, M. *et al.* Superfluid A–B surface tension. *Physica B: Condensed Matter* **329-333**, 122–125 (2003). URL `https://doi.org/10.1016/s0921-4526(02)01919-1`.

17. Bartkowiak, M. *et al.* Interfacial Energy of the Superfluid ^{3}He A-B Phase Interface in the Zero-Temperature Limit. *Phys. Rev. Lett.* **93**, 045301 (2004). URL `https://link.aps.org/doi/10.1103/PhysRevLett.93.045301`.

18. Kaul, R. & Kleinert, H. Surface energy and textural boundary conditions between a and b phases of 3 he. *Journal of Low Temperature Physics* **38**, 539–552 (1980).

19. Schopohl, N. Spatial dependence of the order parameter of superfluid ^{3}He at the A-B phase boundary. *Phys. Rev. Lett.* **58**, 1664–1667 (1987). URL `https://link.aps.org/doi/10.1103/PhysRevLett.58.1664`.

20. Thuneberg, E. V. A-B interface of superfluid ^{3}He in a magnetic field. *Phys. Rev. B* **44**, 9685–9691 (1991). URL `https://link.aps.org/doi/10.1103/PhysRevB.44.9685`.
